# Effects of aroma and taste, independently or in combination, on appetite sensation and subsequent food intake

**DOI:** 10.1016/j.appet.2017.04.005

**Published:** 2017-07-01

**Authors:** Wenting Yin, Louise Hewson, Robert Linforth, Moira Taylor, Ian D. Fisk

**Affiliations:** a The University of Nottingham, Department of Food Sciences, Sutton Bonington, LE12 5RD, UK; b The University of Nottingham, Faculty of Medicine & Health Sciences, Queen's Medical Centre, NG7 2UH, UK

**Keywords:** Appetite, Food intake, Aroma, Taste, Flavour, Cross-modal perception

## Abstract

Food flavour is important in appetite control. The effects of aroma and taste, independently or in combination, on appetite sensation and subsequent food intake, were studied. Twenty-six females (24 ± 4 years, 20.9 ± 1.9 kg⋅m^−2^) consumed, over 15 min period, one of four sample drinks as a preload, followed by an *ad libitum* consumption of a pasta meal (after 65 min). Sample drinks were: water (S1, 0 kcal), water with strawberry aroma (S2, 0 kcal), water with sucrose and citric acid (S3, 48 kcal) and water with strawberry aroma, sucrose and citric acid (S4, 48 kcal). Appetite sensation did not differ between the S1 (water), S2 (aroma) and S3 (taste) conditions. Compared with S1 (water), S2 (aroma) and S3 (taste), S4 (aroma + taste) suppressed hunger sensation over the 15 min sample drink consumption period (satiation) (p < 0.05). S4 (aroma + taste) further reduced hunger sensation (satiety) more than S1 at 5, 20 and 30 min after the drink was consumed (p < 0.05), more than S2 (aroma) at 5 and 20 min after the drink was consumed (p < 0.05), and more than S3 (taste) at 5 min after the drink was consumed (p < 0.05). Subsequent pasta energy intake did not vary between the sample drink conditions. S4 (aroma + taste) had the strongest perceived flavour. This study suggests that the combination of aroma and taste induced greater satiation and short-term satiety than the independent aroma or taste and water, potentially via increasing the perceived flavour intensity or by enhancing the perceived flavour quality and complexity as a result of aroma-taste cross-modal perception.

## 1 Introduction

Over-consumption of food, resulting in excessive energy intake, has contributed to the obesity pandemic. Appetite and food intake in humans are controlled by successive but also overlapping sensory, cognitive, hormonal and metabolic signals that influence eating as described in the satiety cascade (Blundell and Bellisle, 2013, Blundell et al., 1987). Within the satiety cascade, “Satiation” is the process which leads to the termination of eating; “Satiety” describes the inter-prandial period during which the feeling of fullness lingers before hunger returns (Benelam, 2009). Food flavour, an oral-sensory signal, may play an important role in affecting appetite sensation and food intake. On the one hand, flavour contributes to food palatability which has been shown to stimulate hunger and increase food intake (Bobroff and Kissileff, 1986, Hill et al., 1984, Spiegel et al., 1989, Yeomans, 1996). On the other hand, flavour *per se* can also be a satiation cue that reduces meal size, and acts as a satiety cue to influence the size of the next meal, through both psychological and physiological mechanisms (Blundell and Bellisle, 2013, Blundell et al., 1996). The flavour modality, which can comprise of both aroma or taste, has been shown to enhance the sensation of fullness, suppress hunger sensation and reduce food intake (Bolhuis et al., 2011, Ramaekers et al., 2011).

There is an increasing interest in the impact of aroma and odour on appetite sensation and food intake. Volatile compounds can reach the olfactory epithelium through one of two routes: orthonasal (via nostril) or retronasal delivery (via nasopharynx) delivery (Negoias, Visschers, Boelrijk, & Hummel, 2008). Orthonasal odour delivery may be linked to the identification and anticipation of a food reward while retronasal aroma delivery is typically associated with the flavour perception of food during an eating event (Small, Gerber, Mak, & Hummel, 2005). Orthonasal odour delivery has been reported to stimulate a specific appetite for food containing this aroma, and it had a relatively smaller appetising effect on other foods (Ramaekers, Boesveldt, Lakemond, van Boekel, & Luning, 2014). In contrast, retronasal aroma delivery has been shown to enhance the feeling of satiation and reduce food intake. A more intense retronasal aroma profile led to an increased sensation of satiation when compared with a less intense retronasal aroma profile in yoghurt products (Ruijschop, Boelrijk, De Ru, De Graaf, & Westerterp-Plantenga, 2008). The addition of a creamy aroma to the nasal cavity via a silicone tube (mimicking the retronasal delivery), while consuming a tomato soup, enhanced the sensation of satiation compared with the condition without the creamy aroma (Ramaekers et al., 2011). This resulted in a 9% reduction in the *ad libitum* intake in the soup with the delivery of a longer and more intense tomato aroma, compared with the same soup with a shorter and less intense tomato aroma (Ramaekers, Luning, et al., 2014).

There are five detectable tastes by humans, including sweet, salty, sour, bitter, umami and a number of potential other tastes including fatty (Chandrashekar et al., 2006, Mattes, 2009). It is now widely agreed that in humans food intake is controlled by learned satiety (conditioned satiety) whereby we associate the sensation of taste with its metabolic consequences through instinct or learned experiences (Booth, 2009). For instance, the learned association between sweet taste and the ingestion of carbohydrates, or between umami and the ingestion of protein, may contribute to the control of meal size (Chandrashekar et al., 2006, Hogenkamp et al., 2011). Of all taste, the effect of sweet taste on appetite sensation and food intake is the most studied. Nutritive sweeteners, such as sugars, may not only promote satiation and satiety via their sweetness, but also, via physiological mechanisms due to their post-ingestive feedback (Bellisle, Drewnowski, Anderson, Westerterp-Plantenga, & Martins, 2012). For example, sucrose (135 g), when presented in a drink, increased the subsequent feeling of fullness compared with a water control (J. H. Lavin, French, & Read, 2002). Prolonged consumption of sucrose, over 10 min, decreased the subsequent food intake to a greater extent when compared to the condition where sucrose was consumed over 2 min, suggesting that the temporal profile of sweetness perception may modulate subsequent food intake (J. H. Lavin, French, Ruxton, & Read, 2002). A non-nutritive aspartame sweetened drink also suppressed subsequent food intake compared with a water control, but the reduction in food intake after aspartame was smaller than that after sucrose (65 kcal or 90 kcal). This provides the evidence that the post-ingestive consequence of sugars plays a part in providing satiety (Birch, Mcphee, & Sullivan, 1989) although this was in children. Although the sweet taste *per se* can contribute to satiation and satiety, sweet taste often plays a key role in determining the palatability of food or drink which has been shown to stimulate hunger (Bellisle et al., 2012). In some studies, sweet taste had no effect or even increased hunger sensation and food intake, which was potentially due to the palatability from the sweet taste neutralising or even overriding the satiation and satiety signals (Black et al., 1993, Blundell and Hill, 1986, Holt et al., 2000, King et al., 1999).

Flavour perception is the combination of multisensory modalities, of which aroma and taste are the two primary drivers (Auvray and Spence, 2008, Wallace, 2015). Taste or aroma does not only affect the perceived flavour as an independent modality, but the combination of taste and aroma can also change both the intensity and quality of the perceived flavour as a result cross-modal association (Wallace, 2015). Congruent taste and aroma modalities, when presented together, increased the perceived flavour intensity more than the sum of the independent taste and aroma (Hewson et al., 2009, Pfeiffer et al., 2006). Aroma-taste cross-modal association was supported by neural imaging studies. Overlapping areas in the insula, orbitofrontal cortex (OFC), amygdala and anterior cingulate cortex (ACC) have been shown to be activated by taste or aroma modality (Rolls, 2015, Small et al., 1997, Small et al., 2005), whereas a lateral anterior region of the OFC was activated only by the combination of aroma and taste but not by a single aroma or taste modality (de Araujo, Rolls, Kringelbach, McGlone, & Phillips, 2003). Potentially, aroma and taste do not only affect appetite and food intake independently but also as a synergistic combination of both modalities. Warwick, Hall, Pappas, and Schiffman (1993) reported that the combination of vanilla aroma and aspartame in a meal decreased the subsequent hunger sensation, compared with a nutritionally same but unflavoured meal. However, the effect of the combination of aroma and taste modalities, in comparison to the independent effect of aroma or taste modality, on appetite sensation and food intake has not been reported previously, as far as the authors are aware.

The objective of this study was, therefore, to investigate the impact of aroma and taste, independently and in combination, on appetite sensation and subsequent food intake. A flavoured drink model was constructed with different combinations of strawberry aroma and taste substances (sucrose and citric acid). Appetite sensation was evaluated during and after consumption, and food intake at the next meal measured. Sucrose and citric acid may interact with some aroma at a physicochemical level, resulting in changes in the aroma delivery to the nasal cavity. Therefore, the atmospheric pressure chemical ionisation mass spectrometry (APCI-MS) was used to measure any change in the in-vivo strawberry aroma release which may influence appetite sensation and food intake (Taylor, Linforth, Harvey, & Blake, 2000).

## 2 Materials and methods

### 2.1 Study design for evaluating appetite sensation and subsequent pasta intake

The study was a single-blind, randomised crossover experiment. A “preloading paradigm” was used to investigate the effects of aroma and taste, independently and in combination, in a liquid preload, on self-reported appetite sensation and subsequent pasta meal intake. Water without any taste or aroma substances was used as a control preload in parallel to the three sample drinks. This study was approved by the Medical Ethical Committee of the University of Nottingham (ethics reference number: R14032013 SBS Food, 15/03/2013).

### 2.2 Participants for evaluating appetite sensation and subsequent pasta intake

A recruitment email with the inclusion and exclusion criteria was sent to prospective participants. They were asked to participate voluntarily in the study by replying to the email. Male participants were excluded from this study to reduce any variation caused by gender differences in flavour perception, appetite sensation and food intake (Olofsson and Nordin, 2004, Sudo et al., 2004). Inclusion criteria were that participants were 19–40 years healthy non-smoking females, with a normal BMI within 18.5–24.9 kg⋅m^−2^, who were neither pregnant nor breastfeeding, and not taking any medication except the oral contraceptive pills. Exclusion criteria included a weight loss or gain of more than 4 kg in the past six months, self-reported abnormal gustatory and olfactory senses, any allergy or intolerance to the food ingredients, a score >7 for the restraint factor on the Three-Factor Eating Questionnaires (Stunkard & Messick, 1985), or clinically depressed as indicated by a score > 10 on the Beck Depression Inventory (Beck, Ward, Mendelson, Mock, & Erbaugh, 1961).

Respondents were invited to the Sensory Science Centre (SSC) at the University of Nottingham for a screening session. The study was explained to all participants who were given the opportunity to ask questions. All participants signed written informed consent prior to participation. Participants were only informed that this study was about food and appetite. No further information was given to them to prevent response bias. The weight and height of each participant were measured, and they completed the Three-Factor Eating Questionnaires, Beck Depression Inventory, and International Physical Activity Questionnaires (IPAQ) (Craig et al., 2003). The participants who were recruited had a mean BMI of 20.9 ± 1.9 kg⋅m^−2^, a mean age of 24 ± 4 years, a mean restraint score of 4 ± 2 on the Three-Factor Eating Questionnaires, and a mean score of 3 ± 2 on the Beck Depression Inventory.

### 2.3 Procedure for evaluating appetite sensation and subsequent pasta intake

A brief training on reporting appetite sensation using the Visual Analogue Scale (VAS) was given to the 26 selected participants, followed by an in-lab practice prior to the study sessions. Each participant completed all the four sample drink conditions on 4 separate days during their luteal phases (days 18–25 of a menstrual cycle) over 2 to 3 menstrual cycles. This was to minimise the differences in their appetite status across different phases of the menstrual cycle (Hirschberg, 2012, Li et al., 1999). Between any two session days, there was a time interval of at least 3 days. At least 24 h prior to each visit, participants were required to refrain from intense exercise, smoking, alcohol consumption and any medication including the oral contraceptive pills (Astbury, Taylor, & Macdonald, 2011). They were also instructed to eat the same self-chosen dinner on the evening before each session day, between 20.00 and 21.00 h. Participants were then required to fast until arriving in the laboratory the next morning. Water consumption was permitted while fasting.

**Fig. 1:**
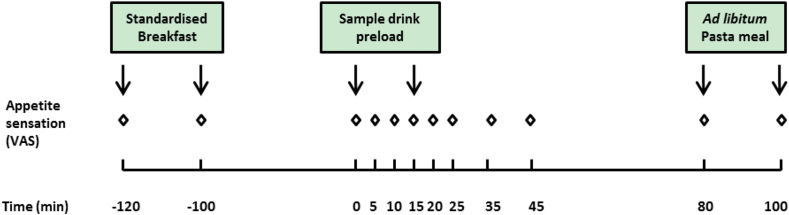
Protocol for each study visit. The sensation of hunger, satisfaction, fullness, desire to eat and the prospective consumption were rated on 100 mm long VAS scales at time points indicated with open diamond markers. The arrows indicate the start and the end of breakfast, sample drink or pasta meal.

On each of the four test sessions, participants arrived at the laboratory at 08.45 h. Participants were requested to remove heavy shoes or clothing, and their body weight and height were assessed. Baseline appetite sensation was reported at 09.00 h using the Visual Analogues Scales (VAS). Each participant was then provided with a standardised breakfast to consume within 20 min. At 11.00 h, participants were provided with one of the four sample drinks to consume over a 15 min period. VAS ratings were completed immediately before (at t = 0 min), and at t = 5, 10, 15, 20, 25, 35, 45, 80 and 100 min after starting to consume the sample drink (Fig. 1). At 12.20 h (t = 80 min), participants were served a pasta meal to consume freely, until they felt comfortably full. Unlimited water was allowed during the pasta meal. No other food or drink consumption was permitted. Participants were required to stay quietly in a waiting room, when not required to undertake study-related activities. All study sessions were conducted in an air-conditioned room at 18 °C, under Northern Hemisphere daylight, in independent booths designed to meet ISO: 8589:2007 (ISO, 2004, p. 16).

### 2.4 Sample drinks for evaluating appetite sensation and subsequent pasta intake

The sample drinks consisted of Evian mineral water (Danone Group, France), Silver Spoon granulated sugar (British Sugar PLC, Peterborough, UK), citric acid (Fisher Scientific, UK) and a multi-component strawberry aroma (Mane Co. Ltd., Derby, UK). Strawberry aroma compounds included ethyl butyrate, ethyl 2-methyl butyrate, and ethyl hexanoate, which were diluted in propylene glycol. All sample drinks were freshly made 2 h prior to serving and were served at 18 °C. Composition and energy content of sample drinks are shown in Table 1.

**Table 1:** Sample drink composition in water and total energy content.

Sample drink	Aroma	Taste		Energy kcal/150 mL
	Strawberry aroma v/v	Sucrose w/v	Citric acid w/v	
Sample 1 (S1)	0.0%	0.0%	0.0%	0
Sample 2 (S2)	0.5%	0.0%	0.0%	0
Sample 3 (S3)	0.0%	8.0%	0.1%	48
Sample 4 (S4)	0.5%	8.0%	0.1%	48

Each sample drink was served in 15 sealed cups coded with a 3-digit random number, each containing 10 mL. It has been shown previously that oral-sensory-induced satiation and satiety appears to develop and increases after a period of intense oral sensory exposure (Bolhuis et al., 2011). Therefore, participants were instructed to consume the sample drinks slowly over 15 min using a straw (diameter: 0.625 mm, Altec Ltd., UK), allowing sufficient oral-sensory exposure to elicit the development of satiation and satiety. Meanwhile, participants were instructed to focus their attention on the flavour perception of the drinks during drinking, as the distraction during an eating process can increase the *ad libitum* food intake (Brunstrom & Mitchell, 2006). Each participant consumed a total of 150 mL of a sample drink over 15 min. While drinking, participants were instructed to keep their normal breathing rate, swallow at a comfortable frequency, and focus on the perception of the flavour.

### 2.5 Pre-visit dinner for evaluating appetite sensation and subsequent pasta intake

On the evening prior to the study, participants were free to choose a dinner within their regular diet, but they were requested to eat a comparable meal of equivalent energy value and type prior to each study visit.

### 2.6 Breakfast

A breakfast of Rice Krispies (Kellogg's UK Limited, Manchester, UK) and semi-skimmed milk (Sainsbury's Supermarkets Ltd., London, UK) was provided to each participant. It was equivalent to 10% of the participant's estimated total daily energy expenditure (TDEE), and it contained 72%, 14% and 14% energy from carbohydrate, protein and fat, respectively (Astbury et al., 2011). TDEE was calculated as the Basal Metabolic Rate (BMR) multiplied by 1.38, 1.55 or 1.73 (activity index) based on the individual's activity level obtained from the IPAQ questionnaire (Craig et al., 2003). Individual's BMR was calculated according to the equation by Harris and Benedict (1919).

### 2.7 Pasta meal

The *ad libitum* pasta meal was made of penne pasta (Sainsbury's Supermarkets Ltd., London, UK), Dolmio Garden Vegetables pasta sauce (MARS Food, USA), olive oil (Sainsbury's Supermarkets Ltd., UK), and cheddar cheese (Sainsbury's Supermarkets Ltd., UK). The penne pasta was cooked and mixed with the pasta sauce, olive oil and cheddar cheese on the evening before each study day, using a standard cooking procedure. The cooked pasta was refrigerated in sealed containers overnight until it was reheated in a microwave oven for 3 min and then stirred immediately before serving. The pasta meal had an energy density of 1.66 kcal⋅g^−1^, and it contained 17%, 62%, 18% and 3% of protein, carbohydrate, fat and fibre, respectively.

### 2.8 Measuring appetite sensation

Participants rated their appetite sensation of hunger, satisfaction, fullness, desire to eat and the prospective consumption (the amount they anticipated they might consume), using a 100 mm long visual analogue scale (VAS) (Flint, Raben, Blundell, & Astrup, 2000) at defined time points across the session (Fig. 1). Each scale was anchored with “not at all” and “extremely” at either end. The question for each appetite sensation was: “How hungry do you feel?” (hunger); “How full do you feel?” (fullness); “How satisfied do you feel?” (satisfaction); “How strong is your desire to eat?” (desire to eat); and “How much do you think you can eat?” (prospective consumption). Participants were asked to score on the scales by placing a mark on the horizontal line, using the computerised data acquisition system FIZZ 2.46 (Biosystems, France).

### 2.9 Evaluation of energy intake from the pasta meal

Subsequent energy intake (EI) from the pasta meal was measured 65 min after finishing consumption of the sample drink. Participants were given a 530 g portion of pasta and were instructed to terminate eating when they felt comfortably full. They were instructed to ask for another portion once the previous portion was finished if required. The pasta meal EI was calculated as the weight (g) of pasta consumed multiplied by the energy density of the pasta (1.66 kcal⋅g^−1^).

### 2.10 Participants for the evaluation of flavour intensity and liking

After the completion of the appetite sensation and food intake measurement, 60 healthy female participants, including the previous 26 participants, from the University of Nottingham were recruited to complete two sensory tests to evaluate the flavour intensity and liking of the previous four sample drinks, on two separate days. In addition, 5 of these participants completed an extra session to measure *in-vivo* aroma release of the sample drinks by APCI-MS. Participants were non-smoking females of normal weight (BMI: 18.5–24.9 kg⋅m^−2^), with no abnormal gustatory or olfactory senses, and no allergy to the ingredients used. Each participant's height and weight were measured. Any participants who had a BMI outside 18.5–24.9 kg⋅m^−2^ were excluded from the study. The study procedure was explained to all potential participants, who were given the opportunity to ask questions. They all signed written informed consent prior to participation. The 60 female participants had a mean BMI of 21.4 ± 2.1 kg⋅m^−2^, and a mean age of 22 ± 4 years (18–36 years).

### 2.11 Pairwise ranking for comparing perceived overall flavour intensity

The four sample drinks were compared for the perceived overall flavour intensity using pairwise ranking test (Meilgaard, Civille, & Carr, 2006). The term “flavour” was explained to participants as the combination of gustatory, olfactory and trigeminal sensations (ISO, 2008). 10 mL of each sample was present in a 30 mL sealed plastic cup coded with a 3-digit random number. Each participant evaluated all six possible pairs formed from the four samples (2 by 2), one pair at a time, with the question “which sample is stronger in the perceived overall flavour intensity”. Sample pairs were presented in a balanced and randomised order between and within pairs. Participants were asked to take a 5 min break after assessing 3 pairs of samples. Water and crackers were used to cleanse the palate between samples.

### 2.12 Hedonic test for liking ratings

The overall sensory liking of the four sample drinks was assessed using a 9-point hedonic scale (Peryam, 1952). Score “9” was assigned to “like extremely”, “5” to “neither like nor dislike”, and “1” to “dislike extremely”. 10 mL of each sample was present in a 30 mL sealed plastic cup coded with a 3-digit random number. All 60 participants assessed the four sample drinks in a randomised and balanced order. Water and crackers were used to cleanse the palate between samples.

### 2.13 APCI-MS analysis of in-vivo volatile release

To test whether the addition of 8% sucrose and 0.1% citric acid affected the strawberry aroma release to the nasal cavity, breath by breath APCI-MS analysis was used to compare the in-nose strawberry aroma concentration between S2 (only strawberry aroma) and S4 (strawberry aroma + citric acid + sucrose). Five participants consumed both samples in triplicate. Participants were instructed to drink 20 mL of each sample in one mouthful while positioning one nostril on the nasal sampling tube of the APCI-MS, breathing and swallowing regularly. Nose breath was sampled at a flow rate of 25 mL⋅min^−1^. The release of key strawberry aroma molecules, ethyl butyrate, ethyl 2-methyl butyrate and ethyl hexanoate was determined by monitoring the m/z of 117, 131 and 145, which are the mass-to-charge ratios for each protonated molecule. The sample drink presentation order was balanced and randomised.

### 2.14 Data analysis

**Fig. 2:**
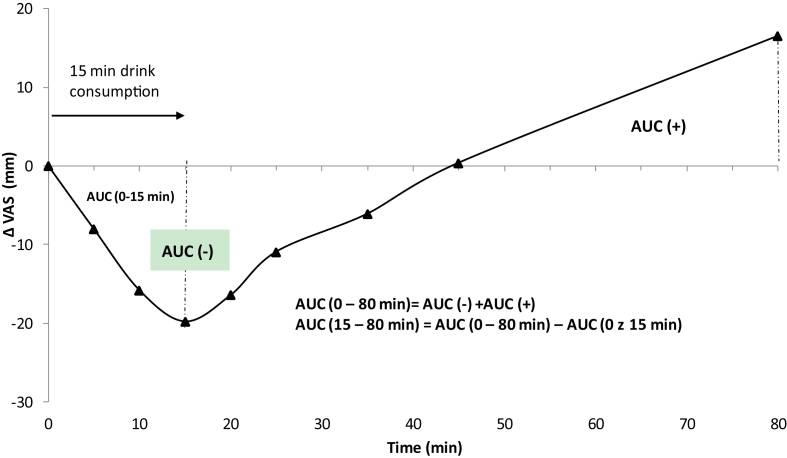
A reference illustration of AUC (+), AUC (−), AUC (0–15 min), AUC (15–80 min) and AUC (0–80 min). AUC (−) is the total AUC under the x-axis, while AUC (+) is the total AUC above the x-axis.

Data is presented as the mean ± standard deviation or standard error. A p-value less than 0.05 was considered statistically significant for all tests. VAS appetite ratings were measured in millimetres from the left end to the points where the participants scored. Since VAS appetite ratings before the sample drink preload (t = 0 min) were not different between the four sample drink conditions, Δ VAS ratings were determined by subtracting the ratings collected immediately before the sample drink consumption (t = 0 min) from the ratings after the sample drink consumption (t = 5, 10, 15, 20, 25, 35, 45, 80 and 100 min). One participant was identified as an outlier whose appetite ratings had residual values above ±3 standard deviations. Data from the remaining 25 participants were used for the final analysis of appetite sensation. The area under the curve (ACU) values for Δ VAS ratings over different time periods was calculated according to the trapezoidal rule. Fig. 2 demonstrates the visual references for the AUC (−), AUC (+), AUC (0–15 min), AUC (15–80 min) and AUC (0–80 min). AUC (−), AUC (0–15 min), AUC (15–80 min) and AUC (0–80 min), pasta meal energy intake (EI) and the accumulated energy intake (sample drink energy + pasta EI) were compared between the four sample drink conditions using one-way repeated measures ANOVA (one factor: sample drink). Two-way repeated measures ANOVA (4 sample drinks × 3 time-points) were conducted to assess the effect of sample drink over the sample drink consumption period (5, 10 and 15 min) on Δ VAS ratings. Additional two-way repeated measures ANOVA (4 sample drinks × 6-time points) were conducted to assess the effect of sample drink over the period after the sample drink was consumed (15, 20, 25, 35, 45 and 80 min) on Δ VAS ratings. Δ VAS ratings between the sample drink conditions was compared at each study time point using one-way repeated measures ANOVA (one factor: sample drink). If a significant main effect of sample drinks was obtained from ANOVA, *post hoc* tests with Bonferroni correction for pairwise multiple comparisons were done to determine which samples were significantly different. Satiation effect of the sample drink (the suppression of hunger over the sample drink consumption period) was evaluated by AUC (0–15 min) and the change in Δ VAS over the sample drink consumption period (between t = 5 min and 15 min). Satiety effect of the sample drink (the suppression of hunger after the sample drink was consumed) was indicated by the value of AUC (15–80 min), Δ VAS ratings over the subsequent 65 min after the sample drink was consumed (between t = 15 and 80 min) and the subsequent pasta meal energy intake (Masic & Yeomans, 2013).

To compare the perceived overall flavour intensities, rank sums of each sample drinks obtained from the pairwise ranking test were analysed using Friedman's test with Tukey's HSD *post hoc* tests (Meilgaard et al., 2006). The rank sum of each sample was calculated by adding the sum of times when a sample was selected as less intense to twice the sum of times when the sample was selected as more intense in the perceived overall flavour. Liking ratings were analysed using Friedman statistic test with *post hoc* Wilcoxon signed-rank test. For APCI-MS data analysis, the peak height values from the chromatograms for each ion from S2 (aroma) and S4 (aroma + taste) were obtained using MassLynx software (Micromass Ltd, UK). The mean peak values of ions 117, 131 or 145 were compared between S2 (aroma) and S4 (aroma + taste) using paired sample t-tests. The objective was only to compare the intensities of aroma delivered from S2 and S4. Therefore, aroma intensity measured as arbitrary units (relative intensity ratio) was sufficient to analyse differences. All statistical tests were performed using IBM SPSS Statistics 21.0 (IBM Corporation, USA).

## 3 Results

### 3.1 Appetite sensation

**Fig. 3:**
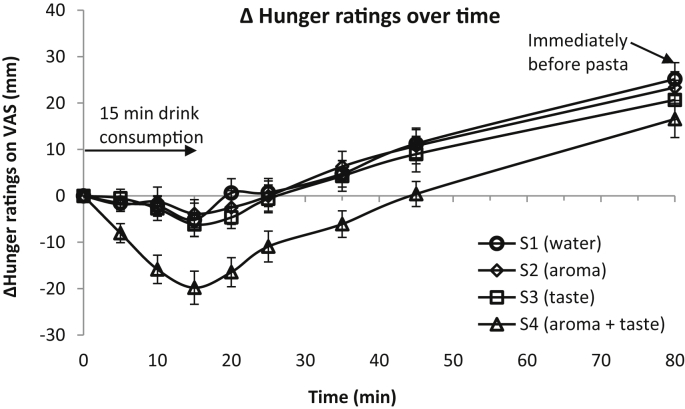
Mean Δ hunger over 5, 10, 15, 20, 25, 35, 45, and 80 min after starting to consume sample drink of S1 (water), S2 (aroma), S3 (taste) or S4 (aroma + taste), n = 25 participants. Error bars represent standard errors.

The VAS appetite sensation for hunger (Fig. 3), fullness, satisfaction, desire to eat, and prospective consumption, at the time point before the breakfast (t = - 120 min) were not significantly different between the four experimental visits, indicating that the participants arrived each time with a similar initial appetite status. There was no significant main effect of sample drink on ratings of Δ fullness, Δ satisfaction, Δ desire to eat and Δ prospective consumption, and their corresponding AUC (0–15 min), AUC (−), AUC (15–80 min) and AUC (0–80 min) values. There was a significant main effect of sample drink on AUC (−), AUC (0–15 min), AUC (15–80 min) and AUC (0–80 min) for Δ hunger values (p < 0.05). Fig. 3 and Table 2 show, respectively, the VAS Δ hunger ratings and the AUC values for Δ hunger over different study periods in each sample drink conditions. S4 (aroma + taste) reduced Δ hunger AUC (−) and Δ hunger AUC (0–80 min) indicating the total reduction and net reduction in hunger sensation by the sample drink over the study period compared with S1 (water) (p < 0.05) and S2 (aroma) (p < 0.001). Δ hunger AUC (−) and Δ hunger AUC (0–80 min) were not significantly different between the S4 (aroma + taste) and S3 (taste) conditions, or between the S1 (water), S2 (aroma) and S3 (taste) conditions.

**Table 2:** Mean ± standard error of AUC values for Δ hunger, n = 25 participants.

Sample drink	AUC (0–15 min)	AUC (−)	AUC (15–80 min)	AUC (+)	AUC (0–80 min)
S1	−32 ± 24^A^	−196 ± 60^A^	737 ± 165 ^A^	901 ± 147^A^	705 ± 181^A^
S2	−25 ± 26^A^	−221 ± 64^A^	692 ± 197 ^AB^	888 ± 180^A^	667 ± 216^A^
S3	−32 ± 27^A^	−302 ± 69^AB^	564 ± 210 ^AB^	834 ± 178^A^	532 ± 224^AB^
S4	−169 ± 32^B^	−614 ± 111^B^	25 ± 168 ^B^	469 ± 100^A^	−144 ± 190^B^

Values within a column without the same capital letter superscript are significantly different (p < 0.05).

#### 3.1.1 Satiation evaluation

The Δ hunger ratings decreased over the 15 min consumption of the sample drink (p < 0.005) (Fig. 3). Δ hunger AUC (0–15 min) which describes the reduction in hunger sensation over the 15 min sample drink consumption period (satiation), was larger in the S4 (aroma + taste) condition than the S1 (water) (p < 0.05), S2 (aroma) (p < 0.001) and S3 (taste) conditions (p < 0.05) (Table 2), suggesting that S4 (aroma + taste) induced greater satiation than S1 (water), S2 (aroma) and S3 (taste). There was a significant main effect of sample drink on Δ hunger ratings over the sample drink consumption period (p < 0.05). Pairwise multiple comparison tests with Bonferroni correction (Table 3**)** revealed that S4 (aroma + taste) reduced Δ hunger ratings more than S1 (water), S2 (aroma) and S3 (taste) at t = 5, 10 and 15 min (p < 0.05). There was no significant difference in AUC (0–15 min) or VAS Δ hunger ratings over the sample drink consumption period (at t = 5, 10 and 15 min) between S1 (water), S2 (aroma) and S3 (taste), suggesting that the individual aroma or taste condition and did not affect satiation differently from the water control. The interaction effect between the sample drink and time was not significant.

**Table 3:** Mean ± standard error of Δ hunger ratings in each sample drink condition, n = 25 participants.

	0 min	5 min	10 min	15 min	20 min	25 min	35 min	45 min	80 min
S1	0	−1.6 ± 1.4^A^	−2.2 ± 2.2^A^	−5.2 ± 3.6^A^	0.6 ± 3.1^A^	0.7 ± 3.0^A^	4.8 ± 2.9^A^	11.2 ± 3.0^A^	25 ± 3.5^A^
S2	0	−1.8 ± 1.5^A^	−1.2 ± 3.1^A^	−3.8 ± 3.0^A^	−2.5 ± 3.6^A^	0.0 ± 3.3^A^	6.3 ± 3.3^A^	10.8 ± 3.8^AB^	23 ± 3.4^A^
S3	0	−0.5 ± 1.9^A^	−2.7 ± 2.6^A^	−6.2 ± 2.6^A^	−4.6 ± 2.4^A^	−0.6 ± 3.0^A^	4.3 ± 3.3^AB^	9.0 ± 3.8^AB^	21 ± 4.2^A^
S4	0	−8.0 ± 2.1^B^	−16 ± 3.1^B^	−20 ± 3.6^B^	−16 ± 3.1^B^	−11 ± 3.3^A^	−6.1 ± 2.9^B^	0.4 ± 2.7^B^	17 ± 4.0^A^

Values within a column without a same capital letter superscript are significantly different (p < 0.05).

#### 3.1.2 Satiety evaluation

The satiety effect of the sample drinks was evaluated by the AUC (15–80 min), the Δ VAS ratings after the sample drink consumption (t = 15–80 min) and the subsequent pasta meal intake. The VAS Δ hunger ratings increased over the subsequent 65 min (t = 15–80 min) period after the sample drink was consumed (p < 0.0001) (Fig. 3). AUC (15–80 min) for Δ hunger was smaller in the S4 (aroma + taste) condition than the S1 (water) and S2 (aroma) conditions (p < 0.05), and was not significantly different from the S3 (taste) condition (Table 2). There was a significant main effect of sample drink on the Δ hunger ratings after the sample drink was consumed (p < 0.05). Pairwise multiple comparison tests with Bonferroni correction revealed that S4 (aroma + taste) reduced hunger sensation more than S1 (water) and S2 (aroma) over the 65 min period after the sample drink was consumed (p < 0.05). Specifically, S4 (aroma + taste) reduced hunger sensation more than S1 (water) at t = 20, 35, and 45 min (5, 20 and 30 min after the sample drink was consumed) (p < 0.05) (Table 3), more than S2 (aroma) at t = 20 and 35 min (5 and 20 min after the sample drink was consumed) (p < 0.05). The difference in Δ hunger ratings between S4 (aroma + taste) and S1 (water), or between S4 (aroma + taste) and S2 (aroma) at t = 25 min, was approaching the significant level (p = 0.08 and 0.07, respectively). The difference in Δ hunger between S4 (aroma + taste) and S3 (taste), over the 65 min period after the sample drink preload, was approaching the significant level (p = 0.076). S4 (aroma + taste) reduced hunger sensation more than S3 (taste) only at t = 20 min (5 min after the sample drink was consumed) (p < 0.05) (Table 3).

At t = 80 min, immediately before the *ad libitum* intake of pasta, values of Δ hunger were not different between the four sample drink conditions. The subsequent energy intake of the pasta meal, served 65 min after sample drink preload (t = 80 min), was not different between the four sample drink conditions (Table 4). The accumulative energy intakes (sample drink EI + pasta meal EI) did not significantly differ between the four sample drink conditions. At t = 100 min, immediately after the paste meal, appetite sensation was not different between the sample drink conditions.

**Table 4:** Mean ± standard deviation (n = 26) of energy intakes from pasta meal, sample drinks and accumulative energy intake of pasta and sample drink in the four sample drinks conditions.

Sample drink conditions	Pasta meal energy intake (kcal)	Sample drink energy intake (kcal)	Accumulative energy intake (kcal)
S1	776 ± 96 ^A^	0	776 ± 96 ^A^
S2	781 ± 75 ^A^	0	781 ± 75 ^A^
S3	759 ± 82 ^A^	48	807 ± 82 ^A^
S4	757 ± 89 ^A^	48	806 ± 89 ^A^

Values within a column without a same capital letter superscript are significantly different (p < 0.05).

### 3.2 Perceived overall flavour intensities of sample drinks

**Fig. 4:**
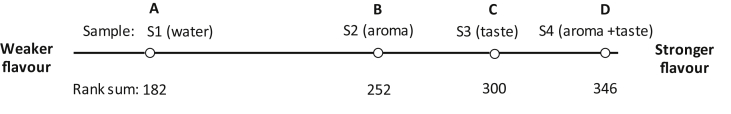
The perceived overall flavour intensities presented as rank sums for the four sample drinks (pairwise ranking test, n = 60). Samples without a same capital letter superscript are significantly different (p < 0.05).

Sample drinks were arranged on a line scale of rank sums of the perceived overall flavour intensity which were calculated from the pairwise ranking test (Fig. 4). A higher rank sum indicates a more intense flavour perception. The perceived overall flavour intensities of the four sample drinks were perceived to be different from each other (p < 0.05). S4 (aroma + taste) was perceived as the strongest in overall flavour intensity, followed by S3 (taste), S2 (aroma), and S1 (water).

### 3.3 Liking ratings

Mean liking ratings (mean ± standard deviation) for S4 (aroma + taste) (6.6 ± 1.5) and S3 (taste) (6.4 ± 1.4) were higher than S2 (aroma) (5.0 ± 0.9) and S1 (water) (5.0 ± 1.0) (p < 0.05). Participants did not like S4 (aroma + taste) and S3 (taste) differently. Their liking ratings for S1 (water) and S2 (aroma) were also not significantly different.

### 3.4 Effect of taste substances on in-vivo strawberry aroma release

There was no significant difference between S2 (aroma) and S4 (aroma + taste) in the in-vivo strawberry aroma release. This indicates that the addition of sucrose and citric acid did not significantly alter the delivery of strawberry aroma to the nasal cavity.

## 4 Discussion

The objective of this study was to investigate the effects of the independent aroma or taste and their combination, in a sample drink, on appetite sensation and subsequent food intake. 26 healthy normal weight female participants consumed four different sample drinks that varied with respect to the presence of aroma and taste stimuli and were subsequently served an *ad libitum* pasta meal 65 min after the sample drink was consumed. Compared with S1 (water), S2 (aroma) and S3 (taste), the drink containing both aroma and taste (S4) induced a greater satiation effect, as indicated by a greater reduction in the hunger sensation over the 15 min sample drink consumption period (t = 5, 10 and 15 min). S4 (aroma + taste) also induced satiety (hunger suppression) more than S1 (water) at 5, 20 and 30 min after the sample drink was consumed (t = 20, 35 and 45 min), more than S2 (aroma) at 5 and 20 min after the sample drink was consumed (t = 20 and 35 min), and more than S3 at 5 min after the sample drink was consumed (t = 20 min). Subsequent pasta energy intake, 65 min after the sample drink was consumed (t = 80 min), did not differ between the sample drinks with an independent aroma or taste stimuli, with the combination of both and the water control.

It is worth mentioning that sucrose was used in this study rather than non-nutritive sweeteners. Non-nutritive sweeteners like stevia, saccharin or aspartame, have secondary taste attributes or aftertastes, such as bitterness and metallic taste (Chandrashekar et al., 2006). Such secondary taste or aftertastes may reduce the palatability/acceptance of the sample drinks, and they are not congruent to the strawberry aroma, both of which are likely to affect the study results and conclusion. Sucrose was chosen as it provided a pure and clean sweet taste that is congruent to the strawberry aroma, however, it contains 4 kcal⋅g^−1^ energy. In the current study, S3 (taste, 48 kcal) did not affect appetite sensation and subsequent pasta meal energy intake differently from S1 (water, 0 kcal) or S2 (aroma, 0 kcal), suggesting neither the energy difference (48 kcal) nor the addition of taste or aroma alone had major impact on appetite sensation and subsequent food intake. Energy in liquid beverages seems difficult to be perceived by the human body and the liquid energy often failed to induce satiation and satiety compared with the energy in solid foods, especially when the energy content is relatively low (Almiron-Roig et al., 2003, Anderson, 1995, Drewnowski and Bellisle, 2007). Participants did not compensate for the small energy difference (48 kcal) between the sample drinks at the subsequent meal. This is in line with previous literature showing that adult participants do not compensate for the energy in a sucrose preload by eating less at the subsequent meal, when the sucrose preload was smaller than 50 g (200 kcal) (Anderson and Woodend, 2003, Anderson, 1995, Birch and Deysher, 1986).

The combination of sugar and citric acid (taste) had no noticeable effect on the self-reported appetite sensation and subsequent food intake. Sugars have been shown to reduce hunger and increase fullness through their sweet taste and energy content (Anderson and Woodend, 2003, Lavin and Read, 2000) and may stimulate hunger and food intake via their enhancement on a food's palatability (Holt et al., 2000). However, little is known about the effects of citric acid and its sourness on appetite sensation and food intake. Further investigations can be done to understand the independent effect of citric acid and the interaction of sucrose and citric acid on hunger sensation and food intake.

Aroma stimuli alone in water (S2) did not affect the self-reported appetite sensation at any time point over the study period and the subsequent pasta energy intake when compared with the water control (S1). Retronasal strawberry aroma delivery has been shown to increase the subjective feeling of satiation, but this effect was observed with the simultaneous delivery of sweet taste from a milk drink (Ruijschop et al., 2008). The retronasal aroma in drink S2 (aroma) was delivered in water without the presence of a noticeable taste stimuli. It may be that retronasal aroma only induces satiation when presented with a congruent taste.

The combination of aroma and taste in a sample drink reduced the sensation of hunger to a greater extent than the independent aroma or taste in a drink over the 15 min sample drink consumption period and until at least 5 min after the sample drink was consumed. One may suspect that an increase in the aroma release from S4 (aroma + taste) as a result of the aroma-taste physiochemical interaction might have contributed to the greater satiation effect (Ruijschop et al., 2008). However, this was not the case in the current study. APCI-MS analysis of in-vivo strawberry aroma release showed that there was no significant difference in the release of strawberry aroma between S2 (aroma) and S4 (aroma + taste). This indicated that citric acid or sucrose did not affect the physical chemistry of the strawberry aroma release. This is in agreement with the current literature that noticeable physicochemical interactions between aroma and taste only appear at relatively high concentrations of both (Friel et al., 2000, Pfeiffer et al., 2006).

The observed difference in hunger sensation between the sample drink conditions does not seem to be caused by the difference in energy content nor the palatability because S3 (taste) and S4 (aroma + taste), sharing the same energy content (48 kcal) and similarly liking ratings, affected the hunger sensation differently. Instead, the difference in the hunger sensation was more likely to result from the difference in flavour perception between the sample drinks. S4 (taste and aroma) was perceived as the most intense in the perceived overall flavour, and it also suppressed hunger sensation more than S1 (water), S2 (aroma) and S3 (taste) over the sample drink consumption period and for a short time after the sample drink was consumed. This suggests that adding the two modalities, aroma and taste, together to a drink reduced the hunger sensation to a greater extent than the independent aroma or taste, potentially via increasing the overall perceived flavour intensity of the drink. Increasing the perceived intensity of a flavour modality (aroma or taste) has been shown to enhance satiation and reduce food intake (Bolhuis et al., 2012, Ruijschop et al., 2008). Increasing the perceived flavour intensity may result in an increase in the overall oral sensory exposure which has been shown to reduce food intake (Bolhuis et al., 2011, de Wijk et al., 2009, Viskaal-van Dongen et al., 2011).

However, the observed greater hunger suppression effect of the combined aroma and taste than the independent aroma or taste, may not only be due to a quantitative increase in the flavour intensity but also due to a qualitative change in the flavour quality and complexity as a result of aroma-taste cross-modal perception. In a previous study, when strawberry aroma, citric acid and sucrose were presented in a drink, participants perceived the flavour of the drink as more intense than the sum intensity of strawberry aroma, citric acid and sucrose presented alone (Pfeiffer et al., 2006). Similarly, in the current study, the combination of aroma (strawberry aroma) and taste (citric acid + sucrose) probably resulted in something more than the sum perception of aroma and taste. APCI-MS analysis showed that there was no significant difference in the release of aroma volatiles from the drink to the nose due to the presence of taste substances (sucrose + citric acid). This suggested that the cognitive process of aroma and taste association, rather than a physiochemical interaction between aroma and taste, might have contributed to the observed hunger suppression. The cross-modal association of aroma and taste results in a more complex flavour perception (Auvray & Spence, 2008). Increasing the complexity of retronasal aroma has been reported to enhance satiation. Participants felt more satiated when consuming a yoghurt with a multi-component strawberry aroma (more complex), compared with the same yoghurt with a single-component strawberry aroma of the similar intensity.

Based on the current study, whether the hunger sensation was reduced as a result of a simple addition of aroma and taste intensities, or due to a change in the perceived flavour quality via cross-modal association is inconclusive. Future experiments could be carried out to further examine this hypothesis. For example, the perceived overall flavour intensity and the palatability of a drink containing a single modality (aroma or taste) could be kept the same as the flavour intensity of a drink containing both aroma and taste modalities, when comparing their effect on appetite sensation and food intake. Alternatively the effect of an incongruent aroma-taste combination on appetite sensation could be studied in comparison to a congruent aroma-taste combination with the similar flavour intensity and palatability. Because the cross-modal association between aroma and taste depends on the congruency of aroma and taste, and non-congruent aroma and taste do not show the same perceptual association that affects the flavour quality.

The mechanism behind the finding that the addition of more flavour modalities reduced hunger sensation is unknown. It may be due to the combination of physiological and psychological mechanisms. Adding flavour modalities may reduce hunger sensation by influencing the hormonal signals for satiation or satiety. Massolt et al. (2010) reported that participants felt less hungry and more satiated after tasting or smelling chocolate compared with the control which involved no tasting nor smelling, and this correlated with a decrease in the blood ghrelin level. In addition, in the current study, participants might be consciously aware of the distinctly different flavour characteristics between the sample drinks. It is likely that participants might have some cognitive belief or expectation about the satiating effect of the sample drinks, which contributed to the difference in appetite sensation, to some extent. The greater reduction in the hunger sensation from a drink with more flavour modalities, hence more intense and complex flavour, might be driven by a learned association (Gibson & Brunstrom, 2007). Participants may have gradually learnt that food with more intense or complex flavour profile may be more nutritionally rich, and therefore, more satiating. Further investigation is required to explain the mechanism behind the study finding and to investigate whether the effects that have been noted are sustained at a similar level over repeated exposure.

Compared with the water control, the sample drink S4 (aroma + taste) reduced the hunger sensation over the 15 min sample drink consumption period (satiation) and continued to suppress the hunger sensation until 30 min (t = 45 min) after the sample drink consumption (satiety). However, this did not result in a significant reduction in the subsequent pasta meal intake. This may be because the difference in appetite sensation ratings was not significantly different between the sample drink conditions immediately before the subsequent pasta meal (t = 80 min). If the time interval between the sample drink preload and the subsequent pasta meal is shortened, a significant reduction in subsequent meal energy intake might occur under the S4 (aroma + taste) condition compared with the water control.

## 5 Conclusion

Taste or aroma stimuli presented alone in a drink preload neither influenced satiation nor satiety, as measured by the appetite sensation and subsequent pasta meal energy intake, compared with the water control. The combination of aroma and taste in a drink induced greater satiation (hunger suppression) than the water control and the drink with the independent aroma or taste. The combination of aroma and taste in a drink briefly further enhanced satiety (hunger suppression) compared with the water control (at 5, 20 and 30 min after the sample drink was consumed), the drink with only aroma (at 5 and 20 min after the sample drink was consumed) and the drink with only taste (at 5 min after the sample drink was consumed). A quantitative increase in the perceived flavour intensity and a qualitative enhancement of the perceived flavour quality as a result of cross-modal association may have contributed to the observed hunger suppression effect of the drink with both aroma and taste. However, subsequent pasta meal energy intake did not differ between the drinks that varied with respect to the presence of aroma and taste stimuli. This study suggests that cross modal flavour enhancement (without the addition of extra energy) may facilitate the development of food or drinks that contribute to the reduction of hunger.
